# Exploring the perspectives of community members on use of Nyaope in Tshwane, South Africa

**DOI:** 10.4102/safp.v65i1.5715

**Published:** 2023-09-28

**Authors:** Doudou K. Nzaumvila, Robert Mash, Toby Helliwell

**Affiliations:** 1 Department of Family Medicine and Primary Health Care, Faculty of Medicine, Sefako Makgatho Health Sciences University, Pretoria, South Africa; 2 Department of Family and Emergency Medicine, Faculty of Medicine and Health Sciences, Stellenbosch University, Cape Town, South Africa; 3 Department of Family Medicine, School of Medicine, Keele University, London, United Kingdom

**Keywords:** community members, Nyaope use, substance abuse, addiction, dependency, Nyaope, community, community engagement

## Abstract

**Background:**

Substance use is a major public health issue in South Africa. Cocktails, containing two or more low-quality substances, have been reported. Nyaope is one of the most popular and is widely available. It has a significant impact on users and communities. The aim of this study was to explore community members’ perceptions of the potential contributors to Nyaope use and dependency.

**Methods:**

This was an exploratory descriptive qualitative study that conducted three focus group interviews with 29 community members. A maximum variation sample was used. Data were analysed using the framework method, assisted by Atlas-ti.

**Results:**

Seven main themes were identified, namely unfavourable home environments, distrust between community members and the local police, easy access to Nyaope at school, inadequate social services, lack of religious or spiritual drive, unfavourable community environments and the effects of Nyaope on users.

**Conclusion:**

The factors identified, were used to construct an emerging model of how Nyaope use is driven in Tshwane. It is clear that a multisectoral response is required involving health and social services, basic education, policing and community leadership. Further research will explore the views of family members and users and quantify the importance of the factors identified.

**Contribution:**

This study showed that rather than a simple linear chain of events, Nyaope use is enabled by a complex system of interconnected elements. According to the respondents, variables in the community at large, the school, the home and the specific user all have a role in Nyaope usage and dependency.

## Introduction

Substance use is a major public health concern in South Africa, being twice as high as the African average.^1^ Cannabis is the most commonly used drug, followed by methamphetamine, heroin and cocaine.^1^ These substances are mostly used in South Africa’s cities and large towns and go by different names in different places, such as dagga for cannabis, crack for cocaine and tik for methamphetamine.^2^ In addition to these drugs, cocktails of two or more low-quality substances have been reported. Since 2000, these have become widespread street drugs in South Africa, primarily in impoverished townships. One of the most popular is Nyaope, which is widely available in Tshwane District.^3,4,5,6^

Nyaope, which means ‘mixture’ in the local language, is a uniquely South African recreational street drug that is also relatively new. From 2000, the use of Nyaope came to be widespread in many townships around Pretoria.^3,4,5,6^ It is considered to be a novel psychoactive substance,^7^ sold in powder form and brown in colour. Its contents vary greatly from batch to batch because it is not manufactured to a precise specification.^8^ As its composition is unknown, Nyaope has not received much attention from the relevant authorities and was not immediately criminalised^9^ although its use continuously escalates and facilitates crime in South Africa.^10,11^ The main ingredient, according to current data, is low-grade heroin. Although antiretroviral medication has been mentioned, current evidence indicates that the cutting agents include cannabis products, milk powder, rat poison, bicarbonate of soda and pool cleaner.^12,13,14,15,16,17,18,19^ Nyaope is rolled with marijuana or tobacco and smoked or injected, usually in rudimentary and unsanitary conditions. In addition, some users inject themselves with blood from another user who is ‘high’ after receiving a Nyaope injection (a so-called ‘bluetooth’ drug).

Nyaope is a highly addictive substance with potentially devastating effects at individual, family and community level.^3,5,6,7^ To service their addiction, the often economically disadvantaged young people using Nyaope struggle financially to fund their Nyaope need. This has wide-ranging effects, such as theft at home or from the neighbourhood, dropping out of school or higher education or criminal activity.^3,10,11,16^ These potentially productive adolescents and young adults become non-functional and live only for their next Nyaope fix.^3,7,14,15,16,17^ Several South African communities are dealing with significant health and social consequences from this addiction.^3,5,6,17^ There have been many types of therapies and attempts to understand the drivers of the phenomenon and to assist users and their families, but current resources and knowledge seem inadequate for sustainable solutions.^18,19,20,21,22,23^ Over 40% of Nyaope users drop out of rehabilitation, with less than 3% of successful recovery among those who complete their therapy.^23^ According to current data, there are insufficient rehabilitation centres in the public sector, while private services are unaffordable for the majority. There are also non-governmental organisations (NGOs) that try to help Nyaope users. Among these is the Community-Oriented Substance Use Programme (COSUP), an organisation that provides services to substance users and their families in 17 centres within Tshwane District.^24^

To address the complex underlying factors that lead to Nyaope use in this community, it is necessary to first understand the perspectives of multiple stakeholders, including the community members, families of users, users themselves and healthcare workers. A series of studies will explore these different perspectives. The aim of this study was to explore the perspectives of community members on the factors contributing to the use of and addiction to Nyaope in Tshwane District.

## Methods

### Study design

This was a descriptive exploratory qualitative study.

### Setting

Tshwane District is in north of Johannesburg in SA’s Gauteng province. Tshwane is divided into seven regions, with 24 townships in total and an estimated population of 2.3 million in 2020.^25^ The municipality has various ethnicities: Afrikaans, English, Sepedi, Sesotho and Xitsonga are among the languages spoken.

In the province, there are currently 12 public rehabilitation centres, three of which are located in Tshwane District. With a total capacity of 65 beds (48 males and 17 females), only two centres can admit patients for all types of substance use.^24^ The Community-Oriented Substance Use Programme, which works as an outpatient programme only, focuses on harm-reduction approaches and clinical care for Nyaope users, rather than on prohibition and abstinence. The Community-Oriented Substance Use Programme offers physical, mental and substance-use screening, assessment, brief interventions and harm-reduction counselling, opioid substitution therapy, needle and syringe exchange, social services, skills development, shelter and referrals to other services.^24^

### Study population and sampling strategy

The study population comprised community members from the townships within the COSUP areas. Purposive maximal variation sampling was used to sample diverse community members in terms of background, employment, profession and residence. The following categories were identified: politicians (community leaders), religious leaders (pastors and priests of different religions), traditional practitioners (sangomas, herbalists and faith healers), police officers, educators (school principals or teachers), healthcare workers (doctors, nurses, community health workers [CHWs]) and others (shop owners or street vendors, as well as taxi owners or drivers). Residents who had been in Tshwane District for less than 12 months, were under the age of 18 years or were known to be current Nyaope users were excluded. Participants were identified by COSUP staff members and recovered users across the 17 COSUP sites and saturation of data was reached after three focus group interviews (FGIs).

### Data collection

The interview guide was based on the risk and protective factors for drug use identified in the National Drug Master Plan 2013–2017^26^ and was aligned with the objectives of the study. Potential risk factors included the amount of unstructured free time, easy access to Nyaope, exposure to substance use in public, neighbourhood affirmation of substance use and a scarcity of job prospects. Protective factors included access to recreational activities and community disapproval of substance use. The opening question was: ‘*Tell us as much as you can about the risk and protective factors you’ve noticed surrounding the use of Nyaope in this neighbourhood*’. We also gave participants the opportunity to express their opinions about any other factors that they felt were not addressed by the interview guide. Open-ended questions were used to explore topics of interest and to allow participants to elaborate on their own issues in their own words.

Focus group interviews were held in the townships of Eesterus, Mamelodi and Shoshanguve. They were facilitated by a nurse and clinical psychologist, who were trained in qualitative interviewing and were fluent with the local languages. The interviewing researcher took field notes and familiarised himself with the collected data. Interviews were audio-recorded and lasted between 60 min and 100 min each. A social worker was available for further counselling or debriefing if required.

### Data analysis

Interviews were transcribed verbatim by a certified linguist, who also translated the transcribed scripts from Sesotho, Setswana, Sepedi and Afrikaans into English. The framework method was utilised to analyse data, which was done in a step-by-step manner as follows^27,28^:

- Familiarisation: The first author began by familiarising himself with the data and field notes taken during data collection.
- Coding index: From there, the first author moved on to the creation of a coding index, which he subsequently organised into categories using an inductive method. The process was supervised by the co-researchers.
- Coding: All transcripts were then coded using the coding index.
- Charting: The researchers then created charts pertaining to the main categories, which brought all the data together.
- Mapping and interpretation: The data were then interpreted to identify themes and subthemes. The range of opinions and experiences within themes was analysed, and any relationships between themes were identified.

*To improve trustworthiness*, the analysis was supported by ATLAS-ti software and conducted by the first author (D.K.N.) under the supervision of R.M. and T.H., who particularly focused on the development of the coding index and interpretation of the data. Although the person who conducted the FGIs (a retired nurse and a psychologist trained in conducting qualitative research) resided in one of the townships afflicted by the Nyaope phenomenon, who had enough interviewing expertise to neither alter nor influence the responses of the participants. In addition, the first author, a family physician, lecturer and researcher had previously worked with Nyaope users in many COSUP centres in the district and in phenomenological research and was well aware of the possibility of bias during data analysis. During data analysis, he was mindful of his own reflexivity by paying attention to his own reactions, ideas and feelings.

## Results

Three different FGIs were needed to reach saturation and included 29 community members (Table 1). Most of the participants were women, over the age of 40 years and from a variety of backgrounds.

**TABLE 1:** Characteristics of participants (*N* = 29).

Characteristic	FGI 1	FGI 2	FGI 3	Total
**Gender**				
Female	7	7	6	20
Male	3	5	1	9
Total	10	12	7	29
**Age group (years)**				
20–29	1	2	0	3
30–39	5	3	0	8
40–49	3	5	5	13
≥ 50	1	2	2	5
**Category of people**				
Educator	2	1	0	3
Community health worker	1	4	1	6
Community leader	1	0	0	1
Nurse	1	0	1	2
Pastor	1	1	1	3
Social worker	2	2	1	5
Police officer	1	1	2	4
Traditional healer	0	1	0	1
Taxi owner or driver	1	1	0	2
Other	0	1	1	2

FGI, focus group interviews.

Seven themes were identified. These were: unfavourable home environment, distrust between community members and police, easy access to Nyaope at school, inadequate social services, lack of religious or spiritual drive, unfavourable community environment and Nyaope’s effects on users. Twenty subthemes were also identified, as shown in Table 2.

**TABLE 2:** Themes and subthemes.

Theme	Subtheme
1. Unbalanced family dynamics	1.1 Poor parenting
	1.2 Parent in denial and/or offers inadequate support
	1.3 Dysfunctional family
2. Poor relationship between communities and police	2.1 Barriers to reporting Nyaope business to the police
	2.2 Barriers to police action
	2.3 Vigilantism, mob justice and parents’ reaction
3. Enablers for Nyaope users in schools	3.1 Availability of Nyaope in schools
	3.2 Inadequate supervision during breaktime
4. Unsatisfactory welfare services	4.1 Poor political commitment
	4.2 Lack of sustainable rehabilitation care
5. Lack of spiritual inclination	5.1 Poor spiritual or religious devotion
	5.2 Demonic possession
	5.3 Laic school curriculum
6. Negative neighbourhood influences	6.1 Supply of Nyaope in the neighbourhood
	6.2 Boredom and idleness
	6.3 Showing off Nyaope as a business gain
7.Nyaope effects on users	7.1 Users are easily hooked
	7.2 Ambivalence in behaviour change

### Theme 1: Unbalanced family dynamics

The participants thought that families of Nyaope users and the home environment were at the core of the matter, with participants often using the phrase ‘it starts at home’.

#### Subtheme 1.1: Poor parenting

Participants blamed the parents of Nyaope users. Parents were described as having been absent from their children’s lives. Participants thought that prior to the use of Nyaope, parents frequently provided inadequate emotional, psychological, intellectual or even physical support to their children. This support deteriorated even more once parents had learned about the use of Nyaope:

‘It all starts at home … The lack of fathers or absent fathers or fathers that are there but they are emotionally detached … they neglect their parental responsibility … I am talking about father-son in particular … of no mentorship.’ (FGI 3, pastor, male)

#### Subtheme 1.2: Parent in denial and/or offers inadequate support

Participants reported that the use of Nyaope in some cases served as a way of coping with family problems. The families of users typically had many other problems, such as family disputes or fights, poor or no communication, and money problems, all of which are further complicated by a lack of or very little discipline. By the time parents became aware of the use of Nyaope, the child had already stopped listening to their parents, and it could be difficult to control them. Worst of all, some parents were in denial, refusing assistance from educators, teachers and others:

‘There should not be compromise with your child’s discipline … You know, but you talk to parents today, specifically mothers, and they say “Mind your own business” … But tomorrow they are quickly here to bail the child out.’ (FGI 2, police officer, female)

Participants agreed that parents of Nyaope users played the most important role, despite the fact that other factors, such as peer pressure, were mentioned as having an impact on Nyaope use and addiction in stable families:

‘So, peer pressure is a contributing factor to Nyaope use … in an unhealthier home environment … unsavoury situation at home, of which finance is a huge factor … lack of cohesion at home … having no sense of belonging, then associate with others from a similar background.’ (FGI 1, pastor, female)

The complexity of the family was the subject of lengthy debates during the FGIs, as some users came from respectable and functional families. Therefore, participants thought that other factors within or outside of the family should be considered:

‘That particular family is doing well financially; the moral support is there, and both parents are available and when you check the siblings, they are attending school and they are doing well, but only this one in particular is using Nyaope … we cannot say that this person does not have support from home.’ (FGI 1, teacher, female)

#### Subtheme 1.3: Dysfunctional families

Parents and siblings noticed the changes far too late when the users were already addicted to Nyaope. To service their habit, users needed cash to buy Nyaope. While the users are still living at home, they start by often repeatedly stealing small amounts of money or objects of poor value, or sometimes very valuable objects of the family members, to sell for much less of their value. This comes at the cost of family stability. Conflicts between the Nyaope user and other family members occur on a regular basis. Such a situation can sometimes become the norm, leading some family members to accept such behaviour (pilfering and stealing at home). This collusion within families, with the understanding that such a situation is normal most of the time by mothers and grandmothers, usually goes against other family members who cannot tolerate the behaviour:

‘My child used to steal from us at home; he would steal from me, steal from his sisters, steal from his father … and his father is a builder. He would steal tools and sell them. He would steal shovels; he was so bad that he even stole wipes, you know the wipes we use for babies … I couldn’t abandon him … His sisters used to shout at me and say I am the one who let him do these things … his father will say let him go with this thing of his.’ (FGI 1, CHCW, female)

### Theme 2: Poor relationship between communities and police

According to the participants, the situation on the ground is unresolvable. When it comes to the Nyaope situation, some participants expressed their despair, disarray and helplessness in relation to any assistance from the police. They did not feel safe cooperating with the police and frequently used the phrase ‘snitching is risky’. Police officers also articulated their powerlessness and inability to carry out any type of operation in the absence of reliable witnesses and community commitment.

#### Subtheme 2.1: Barriers to reporting Nyaope business to the police

Residents are hesitant to report Nyaope dealers for fear of being reported to them by police officers. To put it differently, some participants believed that police officers may be working with or protecting Nyaope business:

‘Snitching is a serious issue. Personally, the police, who work for our government, terrifies me. You will go to them to snitch, and then they will phone the Nyaope dealer and say, “Hey, it’s so and so.” They will come to my house and kill all of us while we are asleep. That is why we are afraid of going to the police station (to report Nyaope dealers).’ (FGI 1, CHW, female)

In some neighbourhoods, Nyaope business is thriving. It is run everywhere in public view to the point where even schools are affected. Community members are fully aware of its effects, but no one has the courage to speak up or report it. Many NGOs have begun to help users by providing them with social support and exchanging syringes for intravenous users, but they must do so cautiously to avoid any sort of conflict with the dealers:

‘If there is a Nyaope dealer in the neighbourhood, no one will say anything. We live next door to them, and they are our neighbours, but no one will point to him and say that’s a Nyaope lord. When COSUP first began, we went around looking for these children in their hotspots. We then ran into a major issue; a social worker who was very active was shot dead and we have no idea who killed her. So, the management told us not to return to the hotspots.’ (FGI 1, social worker 2, female)

#### Subtheme 2.2: Impeded police actions/double-bind

Some police stations are hampered by two factors in their efforts to prosecute Nyaope drug lords and businesses. Firstly, there are no whistle-blowers because of fear of retaliation. Secondly, there are whistle-blowers who inform both the police and the Nyaope dealers simultaneously. In this situation, the police may get criticised for targeting people or premises that are clean of Nyaope when they come:

‘We got a tip from a community member about a house where Nyaope was sold. Then we planned the operation and everything. There was nothing when we arrived … the same individual who informed us likewise informed the dealer … When we arrived, we broke down the doors, and it came back to us that we caused malicious damage, and the owner demanded money from the minister … saying that police officers have damaged his property, accusing him of being drug dealer.’ (FGI 1, police officer 1, male)

This double-bind scenario puts local police stations in a catch-22 situation, as the repercussions make it difficult to take anti-Nyaope measures and social media is also used to highlight their ineffectiveness. To avoid this, the local police must think twice before acting:

‘On that particular day, we fail. We planned, however, to return to that place at some point in the future … in the meanwhile, we are busy with our clandestine operations, believing that at this place we can really come out with something; they are painting us in bad light … people who don’t know anything are making a lot of noise … There are reports on Facebook and Twitter claiming the so-and-so police station is not efficient against Nyaope use.’ (FGI 1, police officer 1, male)

#### Subtheme 2.3: Vigilantism, mob justice and parents’ reaction

Nyaope users require money to satisfy their addiction daily. Although some would rather do odd jobs or any informal work for a pittance, others resort to pilfering, theft from their own households, pick-pocketing, shoplifting, break-ins and robberies in the neighbourhood to get money. The lack of trust in the police leads to some members of the community taking matters into their own hands. Firstly, they use vigilantism as a means of protecting themselves from Nyaope users and, secondly, violence such as assaults or mob justice to punish Nyaope users who are stealing from them:

‘Nearby, Nyaope users have taken over an abandoned house. My home was broken into a couple of months ago. We attended a funeral that day, and when we returned home, the house had been broken into. So many items were taken … After the break-in, I observed that my neighbourhood’s local police wasn’t taking any action against the squatters, so we (community members) evicted them ourselves … you know, I’m a single mother of two girls … the other neighbour is a single mother too with a disabled daughter … What if one of my daughters had been at home on that day? Therefore, we couldn’t continue to wait for the police … we just thought to act.’ (FGI 3, nurse, female)

Some parents purchased Nyaope for their children in an effort to protect them from community assaults:

‘Per day, I could spend R70. I would wrap it up in a plastic and put it somewhere. He used to know the places I used to put the money for him. I was preventing him to steal from other people and get assaulted.’ (FGI 2, CHCW 1, female).

### Theme 3: Enablers for Nyaope users in schools

Some participants spoke out about easy access to Nyaope at schools in their communities, which have become centres for Nyaope use.

#### Subtheme 3.1: Infestation of Nyaope at schools

Some participants reported an unusual increase in the number of learners who use Nyaope on school premises, where they are introduced to it:

‘She stated that she began smoking at school. And it took some time for her parents to realise she was smoking because she smoked at school … they are buying through the fence …’ (FGI 2, social worker, female)

#### Subtheme 3.2: Inadequate supervision during breaktime

It was reported that, in some schools where Nyaope use is booming, pupils are spending extended periods of time without being monitored. These unsupervised times during the day give them opportunity to focus on other activities, such as using Nyaope:

‘She used to smoke at school because at school sometimes they have free periods or the teacher is at the staff room claiming to have lots of work.’ (FGI 2, social worker 2, female)

Nyaope has become part of the daily business at some schools where learners are using it as a recreational activity during free time, and they believe it helps them concentrate or relax and perform better academically:

‘Sergeant X [*police officer*] and I started the school thing that we go to the schools, per classroom … Kids are saying, “Nyaope makes me creative, I can concentrate, I can study.” Others will tell you that it makes them sleepy; it makes them tired.’ (FGI 2, pastor, male)

### Theme 4: Unsatisfactory welfare services

Some participants expressed dissatisfaction with the quality of social services in their communities, which do not address the current Nyaope-use crisis. They blamed it on a lack of political commitment as well as the fact that, in addition to the already existing problem of limited access to rehabilitation centres, there is no post-rehabilitation program.

#### Subtheme 4.1: Poor political commitment

Since Nyaope has been in their communities, many promises have been made, but no prominent changes have been noticed, leading some community members to express their hopelessness for any assistance from local and national politicians. Sporadic actions have been taken from certain departments, but with little coordination between ministries for a long-term solution:

‘The battle against drug dealers is far from over. The nation’s first citizen visited our community twice and showered us with promises.’ (FGI 2, CHCW 2, female)

Although there were a few sporadic government initiatives, there have not been any long-lasting multisectoral actions to combat Nyaope in the community, such as coordination or collaboration between health and social services:

‘They’ll do this after nothing … There is a lack of continuity, and those three important factors – the police, Health Department and Social Development – don’t even cooperate. And they only do so occasionally.’ (FGI 1, community leader, male)

#### Subtheme 4.2: Lack of sustainable rehabilitation care

In addition to the already existing problem of limited capacity in rehabilitation centres around Tshwane, social workers and parents are dealing with relapses. Nyaope users relapse frequently for a variety of reasons, including the lack of a post-rehabilitation program to assist users who return to the same environment where the underlying causes of their Nyaope use remain:

‘I can send people to rehab; they go for six weeks … They come back to the same community, same environment, same unemployment, so relapse is inevitable; there’s nothing else for them to do. So … government if they can step up and step in is to, you know, not a step-up but a step-down process for our heroin, Nyaope users and get them in a skills development programme so that when they come out of rehab, they can have a contribution, something to keep them busy.’ (FGI 3, social worker, female)

### Theme 5: Lack of religious or spiritual inclination

Participants perceived a definite spiritual component to the phenomenon in their respective communities. They reported a poor spiritual or religious devotion, demonic possession and laic school curriculum among.

#### Subtheme 5.1: Poor spiritual or religious devotion

The spiritual context was repeatedly mentioned by participants in expressions such as ‘spiritual disconnect’, ‘empty spiritually’ and ‘void spiritually’. This mention was made not only by religious participants, such as pastors or traditional healers, but also by other participants:

‘There’s a spiritual disconnect with Nyaope use … at schools, I motivate the learners, yet they still have to go back to an unhealthy, uninvolved mother, father … as much church, spiritually, they are not there for the users. They are there for the body, not there spiritually.’ (FGI 2, pastor, male)

Participants tended to agree that a connection to any kind of religion would have produced a different result because it might have prevented users from using Nyaope:

‘Imagine a child stabbing a classmate with scissors. Do you still believe it is solely because he is a Nyaope user? The issue can be decorated in our hearts and minds as we are sitting here, but I still maintain that those people [*Nyaope users*] are spiritually void.’ (FGI 1, teacher, female)

A problem can always be interpreted spiritually because most Africans are believers and spiritual linkages to various aspects of society are strong and vibrant in many communities:

‘We are Africans [believers]. We are accustomed to praying at the start of any gathering. Nyaope users appear to be spiritually void. And we have a tendency to overlook that aspect.’ (FGI 1, community member, female)

Participants expressed their regret that when it comes to Nyaope, there is a tendency to focus more on the medical and criminal aspects, and spirituality is considered as neither a cause nor a potential part of the solution to the problem:

‘We have traditional leaders, religious leaders and leaders from all walks of life … our children (Nyaope users) are spiritually void and nothing is being done to fill that void.’ (FGI 1, traditional healer, female)

#### Subtheme 5.2: Demonic possession

Some participants strongly believed that Nyaope users were demon-possessed because of their strange behaviour, which could not be attributed to a physical origin. Any kind of solution that did not address the spiritual cause would not work:

‘I realised that the intelligent boy was under the influence of demons because I couldn’t understand why he was acting in that way, and I prayed that Satan be cast out of him.’ (FGI 1, teacher, female)

#### Subtheme 5.3: Laic school curriculum

Some participants believed that the current curriculum in public schools, which does not include any religious teaching, may have an impact on pupils’ spirituality and morals. Because of government’s secular nature and the separation of the state from religious institutions, the National Policy on Religion in Education prohibited religion from public schools:

‘Biblical studies, which was called Bibs, I did it from primary until Standard 7 [*Grade 9*] and it was so good … I so wish our government, especially the Department of Basic Education … they need to review their curriculum … I wish they could add something that has more to deal with the children’s morals as a subject.’ (FGI 1, social worker 1, male)

### Theme 6: Negative neighbourhood influences

The availability of Nyaope in some communities was associated with boredom and unemployment, as well as the display of benefits from selling Nyaope, and led some participants to believe that the Nyaope business would flourish forever.

#### Subtheme 6.1: Supply of Nyaope in the neighbourhood

Nyaope is available in many communities, with vendors operating in plain sight and with the knowledge of everyone, including parents, police officers and teachers, during day and night. It is available in the busiest places, such as taxi ranks, train stations and streets. It is reasonably priced and can be found almost everywhere in public. In some areas, young people even gather in the streets and are not afraid to smoke it in public:

‘Nyaope is easily accessible in the neighbourhood … like buying bread and milk.’ (FGI 2, CHCW 2, female)

‘Because it’s cheap … you find it in every corner.’ (FGI 2, CHCW 4, female)

In some places, during recreational activities, children were found smoking a ‘hubbly’, which allows a concoction of multiple substances to be vaporised and inhaled. Unfortunately, many fall into this trap and do not know that Nyaope has been put in it:

‘They smoke it vapourised … They mix Nyaope or weed in there … so in this thing you can mix everything … for a greater kick … That’s where, again, I say parents come in. How can you allow a child to have this in your house? So, you do get parents that will tell you, “I know it’s my child.”’ (FGI 3, pastor, female)

#### Subtheme 6.2: Boredom and idleness

Participants pointed out that boredom and unemployment in their communities were factors that may contribute to people using and becoming addicted to Nyaope:

‘Specifically, Nyaope, it could be … I think boredom as well.’ (FGI 2, CHCW 3, female)

‘I think unemployment is the lead reason for people taking Nyaope. Some of them are well educated and smart and intelligent, but they ended up in the streets because of lack of jobs and they want to make money. When they are marshalling around, someone will come and say, “Take this, it will make you happy.” And they fall for a trap; that is when they start taking Nyaope.’ (FGI 2, social worker 2, female)

#### Subtheme 6.3: Showing off Nyaope as a business gain

Participants expressed their disappointment with the thriving Nyaope industry. Furthermore, dealers tend to flaunt their wealth and lavish lifestyle. This ostentation attracts young people from the economically disadvantaged poor families:

‘The issue is that prevention will be difficult because, according to residents of the township, these boys are unemployed. Nyaope dealers are well off, wealthy and possess this and that … What’s even more tragic is that I asked a 14-year-old boy the other day what he wanted to be or become when he grew up. “A Nyaope dealer,” he added. I then asked him why. “They always have money,” he claimed. “I want to be a Nyaope dealer because they always have money, drive nice cars, and have large homes, whereas sometimes there isn’t bread or food in my house.” Therefore, those are the role models our kids are looking for.’ (FGI 3, pastor, male)

### Theme 7: Nyaope effects on users

Some participants reported that Nyaope is highly addictive, with users easily falling prey to it and having difficulty committing to change.

#### Subtheme 7.1: Users are easily hooked

Participating social workers from COSUP who had been assisting Nyaope users described how quickly and easily their clients became addicted to the substance:

‘You can become addicted after just one puff or injection of Nyaope, the very first time. One female three years ago, when I registered her, she wasn’t using Nyaope. She only used CAT, but her boyfriend used Nyaope. After being introduced by her boyfriend, she became addicted the first time.’ (FGI 1, social worker 2, female)

‘Like this 63-year-old who claimed to have smoked weed his entire life. After receiving some puffs from a friend who was high, he said the feeling was pleasant. The next time he smoked the usual weed, he didn’t like it anymore. So, he asked that friend to provide him with some more of that weed, only to learn that it was actually Nyaope to which he was hooked. He had developed a physical dependence and addiction.’ (FGI 2, social worker, female)

#### Subtheme 7.2: Ambivalence in behaviour change

It was believed that users had little commitment or motivation to stop and were not consistent in their attempts to stop smoking because of the physical dependence on Nyaope:

‘There is a guy who has been on our programme for three years and is still not using methadone. He doesn’t show up for his follow-up appointments. He arrives, we complete our tasks for the day, and then we schedule a time for him. Then he disappears for a further three to four weeks before returning. We now have to start from scratch. There isn’t any consistency.’ (FGI 3, social worker, female)

## Discussion

**FIGURE 1:**
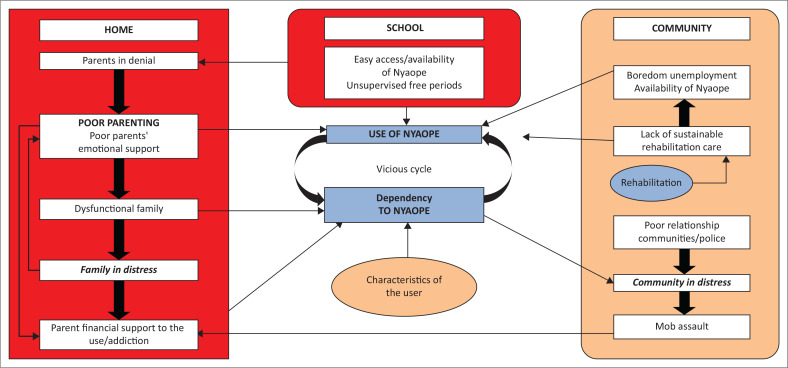
Emerging model of factors underlying Nyaope use.

The key findings of the study are summarised in Figure 1. This study highlighted a complex system of interlinked factors that enable Nyaope use rather than a simplistic linear chain of events. Participants believed that Nyaope use and addiction were related to factors in the community in general, the school, the home and the individual user.

Many homes are devastated by the use of Nyaope.^3,6,19,20^ This study suggested, however, that family dysfunction and ineffective parenting may be a root cause as well. Participants felt that prior to the use of Nyaope, parents might have provided insufficient emotional, psychological, intellectual or physical support to their children. This perceived lack of support may be followed by what was seen as insufficient discipline and, later, a lack of resilience may lead users to succumb to Nyaope in school or on the street. This perceived lack of support may also explain why some community members claim that parents only notice their children are using when they are already addicted or exhibit unusual behaviour. Our participants’ perspectives are consistent with findings in other studies, which identified a lack of family support as one of the possible causes of relapse after Nyaope rehabilitation.^19,21^

Other scholars have confirmed that users commit pilfering from households, burglary, house-breaking and shoplifting to obtain cash to service their addiction.^3,7^ At home, this dishonesty and stealing by users lead to conflict between family members over how to react and contribute to family dysfunction.^19^ Parents have to protect their children against other siblings and household and community members to the extent of even buying Nyaope for them. The findings of this study are consistent with other literature on Nyaope that reports on the paradoxical support of parents for their children’s addiction.^19,21^ In addition to the actual risk of community vigilantism, which may turn into mob justice, some parents might feel abandoned because they lack support. They might feel that by buying Nyaope for their child in this situation, they are protecting them.

We have identified three community factors that drive the problem of Nyoape: poor relationship between communities and police, enablers for Nyaope users in schools and unsatisfactory welfare services. Although Nyaope has been around for more than two decades, it was criminalised only recently.^9^ There is currently more data about crime associated with the use of Nyaope^10,11^ than on the cooperation between the police and communities in which Nyaope is openly used, cheap and easily accessible.^3,22^ The distress felt by communities at the insurmountable problem of Nyoape use and the impunity of dealers has led them to find their own solutions, including community assaults and vigilantism.

The lack of post-rehabilitation aftercare has been noted by others and how this contributes to a vicious circle of rehabilitation and relapse.^21^ There is also mounting evidence of poor social services in general, which is consistent with our data analysis.^3,4,5,6,7,17,18,19,20,21,22^ The failure of health and social services to tackle the problem contributes, in turn, to Nyaope-related family and community distress and dysfunctional solutions.

The presence of Nyaope in schools is not new.^11^ This study suggests that there is easy access for school children and a lack of supervision that allows learners to develop an addiction while at school. It appears that teachers only notice the problem when academic performance plummets and students drop out of school.^23^ Schools should be a safe environment and are an obvious target for more effective prevention and early-detection measures.

Religious or spiritual support was also seen by participants as lacking. One can wonder whether faith-based leaders and traditional healers are more likely to help. Research on Nyaope use, spirituality and religion is scarce.^21^ However, local newspapers and literature on other substance-use disorders have suggested an association between longer term abstinence to substances and increased spirituality and called for further exploration of a link between spirituality, religion and substance use.^29,30,31,32^ This should be given more attention in South Africa, as religion is a strong part of communities, particularly in poorer areas.^33^

It was suggested that users became easily addicted to Nyaope and found it difficult to commit themselves to rehabilitation. Social workers have found users to be dishonest and unreliable when attending rehabilitation.^19^ Relapse and unreliability are issues in all addictions, and in the case of Nyaope, it is driven by the effects of heroin^6,8,12,13^ and other components such as efavirenz, which is known to have psychological effects.^34^ Nyaope has also been shown to cause atrophy of the cerebral grey matter, mainly in regions involved in impulse control, decision-making, social and self-perception and working memory.^5^

### Strengths and limitations

The factors influencing use of Nyaope as found in this study were based on the opinions of community members and as such should be regarded as their subjective truths. Their perspective will be triangulated with that of others in future studies of users and family members. Although saturation of data was achieved, it is possible that some viewpoints and perspectives were missed in the sampling of community members. The findings may be transferable to similar communities in South Africa that have the same dynamics and context.

### Recommendations

This study has identified a set of factors and an emerging model of how these interact to enable the problem of Nyaope use in the community. Further qualitative data need to be collected from family members and users of Nyaope to explore this model and triangulate findings. These studies will contribute to the development of a questionnaire, to be validated by a panel and used in an observational analytical unmatched case-control study to quantify the association of these factors with Nyaope addiction.

The challenges of Nyaope use and addiction need to be urgently addressed and will require a robust multisectoral strategy involving numerous stakeholders, policy change and creative action. Issues that need to be tackled by health and social services include better support for families, particularly post-rehabilitation. Schools need to look at their approach to supervision, safety and early detection of Nyaope use. More robust policing is required, particularly around schools.

## Conclusion

This study has identified factors that community members in Tshwane District believe are driving the use of Nyaope. These factors relate to family function, the role of schools, community distress and vigilantism, as well as the addictive and psychological properties of Nyaope itself. The pervasive sense of helplessness in the face of this problem needs to be tackled through concerted multisectoral collaboration and action. Health, social services, police and basic education must all be involved. Further research will continue to explore the emerging model of factors driving the Nyaope problem and to evaluate which are the most important factors.
